# Neutrophil-Lymphocyte Ratio Predicting Case Severity in SARS-CoV-2 Infection: A Review

**DOI:** 10.7759/cureus.29760

**Published:** 2022-09-29

**Authors:** Sonal Agarwal

**Affiliations:** 1 Department of Pathology, Mahatma Gandhi Hospital, Rajmata Vijaya Raje Scindia Medical College, Bhilwara, IND

**Keywords:** bacterial peptides and proteins, bacterial proteases, neutrophils to lymphocytes ratio, viral infection, proinflammatory cytokines

## Abstract

The severe acute respiratory syndrome coronavirus 2 (SARS-CoV-2) is highly contagious and has taken an enormous toll on the worldwide quality of life and the global economy, in addition to the lives lost due to coronavirus disease 2019 (COVID-19). Precautionary measures and timely identification of the infected cases are essential to minimize the spread of SARS-CoV-2. Infection with this virus causes a spike in the proinflammatory cytokines, resulting in immune system-mediated host tissue damage, thus leading to mortality. Therefore, identifying mild, moderate, and severe cases is crucial to rendering appropriate care. Recent research has focused on identifying laboratory techniques to predict the case severity and outcome of COVID-19 cases. Low serum lymphocyte levels, low lymphocyte-to-C-reactive protein ratio, low platelet-to-lymphocyte ratio, thrombocytopenia, and high neutrophil-lymphocyte ratio (NLR) have been observed in critical infections. NLR might be a prognostic marker for disease severity. Severe cases can be triaged at hospital admission for proper treatment planning and to reduce mortality. This review highlights the potential role of NLR hematological assay in SARS-CoV-2 infection and the mechanism of neutrophilic-induced host tissue damage.

## Introduction and background

The beginning of the 20th century brought three new devastating coronaviruses that affected the entire world. The emergence of severe acute respiratory syndrome coronavirus (SARS-CoV) in 2002, Middle East respiratory syndrome-related coronavirus in 2012, and SARS-CoV-2 in 2019 led to influenza-like symptoms in humans with severe fatal outcomes [1]. Reportedly originating from bats, the coronaviruses transmitted to intermediate animal hosts [2]. With repeated replication, new variants emerged that were capable of transmission to humans [3]. Transmission of coronavirus disease 2019 (COVID-19), the disease caused by SARS-CoV-2, predominantly occurred in a nosocomial manner by droplet infection or close personal contact. Initial clinical presentation was fever and dry cough that could progress to unilateral/bilateral pneumonia, severe acute respiratory distress syndrome (ARDS), acute cardiac injury, renal failure, multiple organ failure, and death [4]. Reverse transcriptase-polymerase chain reaction confirmed the diagnosis, followed by high-resonance computed tomography (CT) of the chest. Various hematocytometric investigations can reveal high neutrophils, low lymphocyte levels, and high platelet levels in SARS-CoV-2 infections. Cao et al. noted low serum lymphocyte levels in COVID-19 cases [5]. Low lymphocyte-to-C-reactive protein ratio, low platelet-to-lymphocyte ratio, and thrombocytopenia have been observed in critical SARS-CoV-2 infections [6,7]. Patients with severe COVID-19 cases had a higher neutrophil-lymphocyte ratio (NLR) ratio than the milder cases, suggesting that NLR might be a prognostic marker for assessing the disease severity [8]. Thus, severe cases could be identified at the time of hospital admission and triaged for proper treatment planning and reduction in mortality.

Studies report cytokine storm and immune dysfunction in COVID-19 cases, with lower lymphocyte and helper T cell counts [9]. There is a slow rise in lymphocytes during recovery [10]. Thus, lymphocytes have limited use in evaluating COVID-19 severity. However, NLR consistently rises early in the disease course, making it a valuable metric. Various preliminary studies have investigated the role of NLR in assessing the severity of other diseases such as asthma [11,12]. This review highlights the potential role of NLR hematological assay in SARS-CoV-2 infections to determine COVID-19 severity and the mechanism of neutrophilic-induced host tissue damage [12].

## Review

Neutrophils in health and disease

Earlier it was thought that neutrophils are a homogenous population with a short life cycle. Lately, heterogeneous populations of neutrophils have been described in infections, autoimmune diseases, and cancers [13]. Pro-neutrophils differentiate into lineage-committed precursors, immature and mature neutrophils. Three distinct subsets of neutrophils have been identified (i.e., homeostatic, aged, and interferon-stimulated gene-related neutrophils) [14,15]. With the expression of C-X-C motif chemokine receptor 2 (CXCR2), mature neutrophils migrate from the bone marrow into blood vessels [16]. Peak neutrophil sequestration occurs at night following a circadian rhythm. With the upregulation of C-X-C motif chemokine receptor 4 (CXCR4) chemokine expression, aged neutrophils migrate back to the spleen, bone marrow, or liver, where they are phagocytosed [17].

Being a part of innate immunity, neutrophils function as the first line of defense. Abnormalities in immune regulation can lead to extensive host tissue damage by these granulocytes. They phagocytose bacteria and kill them by fusing with cytoplasmic granules (i.e., oxidative burst). The cytoplasmic granules contain proteases, defensins, antimicrobial peptides, and reactive oxygen species. The chemokine expression of C-X-C motif chemokine ligand (CXCL) 1, 2, and 8 guides neutrophils' migration and activation. Other inflammatory components such as complement 5a and activated platelets cause the localization of neutrophils and the formation of neutrophil extracellular traps (NETs) [17]. NETs are web-like structures of deoxyribonucleic acid (DNA) and proteins expelled from the neutrophil that ensnare pathogens. The neutrophil platelet aggregation leads to a cascade of events, from the formation of fibrin mesh to trapping the pathogens and NET-induced killing in associated areas [18].

Neutrophils and thrombi formation

Increased vascular permeability leads to proteinaceous exudates in alveolar spaces and pulmonary edema in severe inflammation. Proteinase 3 disrupts the tight junctions, and neutrophil seepage occurs through endothelial cells. Mechanisms of NET formation are not fully understood. Some potentially important enzymes involved in forming these extracellular traps are neutrophil elastase, peptidyl arginine deiminases (PAD), and gasdermin D [19]. PADs are essential in the formation of NETs as they are expressed in granulocytes and mediate histone citrullination. Thus, chromatin decondensation takes place and chromosomal DNA is expulsed with various antimicrobial peptides triggering the NET formation. Excessive exaggeration of these traps can trigger a cascade of inflammatory events that cause collateral damage to the host, formation of microthrombi, irreversible lung damage, and cardiac and renal tissues, apart from having beneficial effects [20]. Lung, heart, and kidney tissue are targeted by SARS-CoV-2 and play an important role in COVID-19 mortality [21].

NET disrupts the granules, releasing histones (present in NET chromatin) and granule proteins such as myeloperoxidase, neutrophil elastase, and proteinase, denoting the epithelial lining and causes platelet aggregation and thrombosis [22]. Elevated levels of extracellular histones have been found in the extracellular lavage and plasma of ARDS patients. The toxicity of naked histones has been shown in various studies [23]. Zuo et al. detected elevated NET breakdown products in the serum of severe COVID-19 patients [24]. Histones also act as ligands for toll-like receptors on platelets and activate them [25]. The interaction of histones and platelet phospholipids activates the coagulation pathway [26].

**Figure 1:**
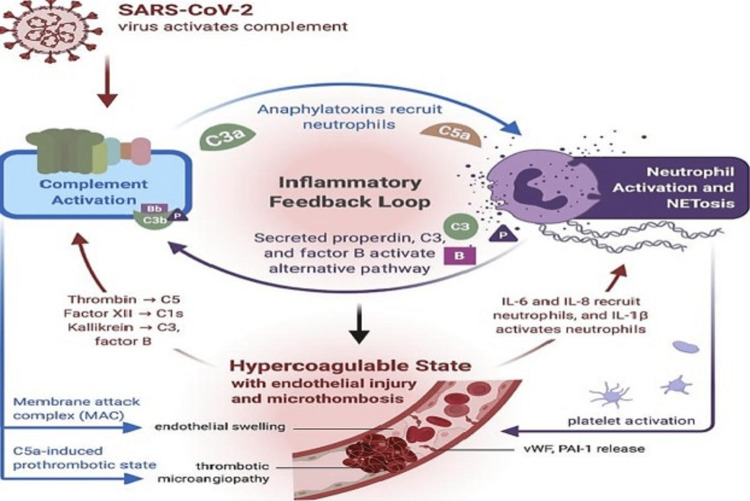
NET and COVID -19 disease Image reproduced from: Java A, Apicelli AJ, Liszewski MK, Coler-Reilly A, Atkinson JP, Kim AH, Kulkarni HS. The complement system in COVID-19: friend and foe? JCI Insight. 2020 Aug 6;5(15):e140711. doi: 10.1172/jci.insight.140711.

NET has been associated with thrombi formation in the arterial-venous system with potentially fatal outcomes. Thus, when NETs circulate at high levels, a significant source of enzymatic activities can exaggerate the small-vessel occlusion. The activation of platelets can lead to enhanced NET formation by neutrophils in a vicious cycle during the patient's clinical deterioration leading to ARDS. Also, neutrophils can phagocytose antithrombin III and tissue factor pathway inhibitors [27]. Animal studies demonstrated that DNAase I dissolves the NET and reduces the thrombosis, improving the perfusion of the cardiac and renal vasculature. Therefore, NET can be a potential target in COVID-19 therapeutics (Figure 1) [28,29].

Neutrophils and the cytokine storm

The severity of clinical conditions in COVID-19 cases has been repeatedly linked to cytokine storms. There is an increase in serum levels of interleukin (IL) 1β, IL-2, IL-6, IL-7, IL-8, IL-10, and IL-17 [30]. Also, serum levels of macrophage inflammatory protein 1α, tumor necrosis factor-α, interferon (IFN) γ, IFN-γ-inducible protein 10, and granulocyte colony-stimulating factor and monocyte chemoattractant protein 1 (MCP1) are elevated [31].

The neutrophilic NET formation, in turn, induces macrophages to increase the expression of IL-1β [31,32]. Thus, uncontrolled inflammatory reactions are achieved by continuous mutual induction of macrophages and neutrophils. Lachowicz-Scroggins et al. established an association between neutrophils and IL-1β in severe asthma patients, in whom COVID-19 can lead to progressive worsening of respiration decompensation and abnormal immune responses [33].

**Figure 2:**
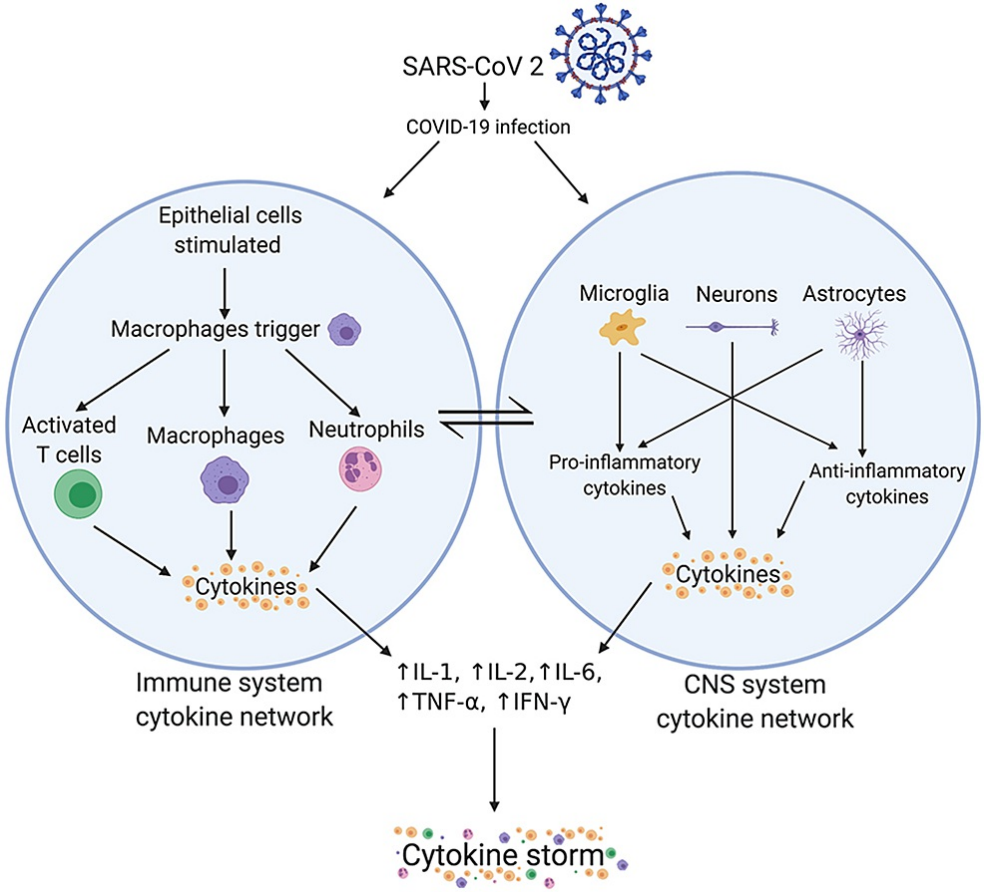
Netrophils and cytokine storm Image reproduced from: Bhaskar S, Sinha A, Banach M, Mittoo S, Weissert R, Kass JS, Rajagopal S, Pai AR, Kutty S. Cytokine Storm in COVID-19-Immunopathological Mechanisms, Clinical Considerations, and Therapeutic Approaches: The REPROGRAM Consortium Position Paper. Front Immunol. 2020 Jul 10;11:1648. doi: 10.3389/fimmu.2020.01648.

IL-8 can also induce neutrophils to release IL-6 receptor (R) α. Another potential target in COVID-19 therapeutics can be IL-6 induced by IL-1β (Figure 2) [34]. Calabrese and Rose-John demonstrated classic and trans-signaling mechanisms for IL-6. IL-6 interacts with IL-6Rα, and gp130 (a cytokine receptor) showed improved lung function in asthma patients with decreased plasma levels of IL-6Rα [35].

Lymphopenia in SARS-CoV-2

The progression of the course of the disease leads to lymphopenia. The SARS-CoV-2 virus targets T lymphocytes, which is significant for a patient with critical conditions. CD8+ subsets of T cells show a marked decrease in the sera of patients admitted to the intensive care unit (ICU). Several studies have reported a close association between cytokine storm and reduction in levels of T cells [36,37]. Higher serum IL-6 levels have been associated with lower T lymphocytes and disease severity. Pathogenesis of lymphopenia in COVID-19 can be attributed to the inflammatory cytokine storm. A correlation has been observed between serum level of pro-inflammatory cytokines such as TNF-α and IL-6 and lymphopenia. Autopsy studies have noted severe lymphocyte destruction correlating with increased serum levels of IL-6 as well as Fas-FasL interactions. Studies reported impaired cytotoxic activity of natural killer (NK) cells and T cells significantly correlated with serum IL-6 levels [36]. Tocilizumab (an IL-6 receptor antagonist) showed promising results with significantly increased circulating lymphocytes [37]. Another mechanism can be T cell exhaustion in SARS-COV infections. Programmed cell death protein 1, T cell immunoglobulin, and mucin domain 3 are the markers of T cell exhaustion. These markers' increased cell surface expression was observed in CD+4 and CD+8 T cells in SARS-CoV-2 infections. Another suggested mechanism could be interference with T cell expansion. Decreased expression of CD107 and IFN-γ (T cell activation markers) was correlated to the severity of the disease, independently to several regulatory T cells. Downregulation of genes (MAP22K7, SOS1) associated with activation of T cell and function has been suggested in some studies. After recovery, gene expression returned to normal levels [38].

NLR as a prognostic tool

An immediate assessment of high-risk patients is needed after diagnosing SARS-CoV-2 infection. SARS-CoV-2 leads to an exaggerated immune response and significant damage to the host tissue by a cytokine storm. The rise in serum IL-1B, IFN-γ, IP10, and MCP1 has been reported. The decrease in CD+4 cells and CD+8 cells, and increased proinflammatory cytokines lead to even more significant lymphopenia. This correlates with severe clinical manifestation, immunosuppression, and NLR increase. Earlier studies have used NLR as a prognostic marker in diseases such as solid tumors, and lung, cardiovascular, and kidney diseases. Therefore, NLR can be used effectively as a simple, cost-effective systemic inflammatory marker in laboratory investigations [39].

Ma et al. suggested that NLR can significantly differentiate severe disease from mild disease [40]. Wang et al. assessed the accuracy and sensitivity of NLR in detecting the severe cases at the time of hospital admission in a meta-analysis. They included 5,570 COVID-19 cases, including 1,607 severe cases. The sensitivity and specificity of NLR were 0.82 and 0.77, respectively. They concluded that NLR could accurately distinguish severe cases of COVID-19 from less severe cases [41]. NLR is a simple, cost-effective hematological tool. In another meta-analysis, NLR was reported to predict severe SARS-CoV-2 infections with accuracy [42]. Heterogeneity in the studies should be included to eliminate geographical and ethnic bias. Also, in most studies, the median patient age was 60 to 70 years. With age, the thymus function declines to approximately one-tenth of a younger adult, with progressive decline in the humoral and cellular immunity. Older patients are at a higher risk of severe infections and mortality than younger patients [43]. Singh et al. analyzed 201 laboratory-confirmed cases of SARS-CoV-2 and divided them into severe and non-severe groups based on clinical manifestation, shortness of breath, and oxygen saturation <93% with a ratio of arterial oxygen partial pressure to fractional inspired oxygen of ≤300 mmHg. NLR on day 1 and day 3 was 7.36 and 7.73, respectively, compared to NLR of 10.8 and 9.11 in the severe group. The overall sensitivity and specificity of NLR on day 1 were 60% and 55%, respectively. The disease progression and mortality rate was 4.4 times higher in the severe group than in the control group. NLR is a time-sensitive variable, and values are dynamic [44]. This supports another study that reported NLR was an independent inflammatory risk factor for mortality during hospitalization. NLR can accurately help identify severe cases early and allow timely intervention to reduce fatality. The results also reinforce the association between NLR and etiopathogenesis of SARS-CoV-2 [45]. Another study associated age and NLR to predict poor clinical outcomes and observed that patients ( aged 50 or older) with NLR > 3.13 developed severe symptoms, while patients( aged 50 or older) with NLR < 3 and young patients showed better clinical outcomes than those admitted to the ICU. In a retrospective study of 74 laboratory-confirmed SARS-CoV-2 infections in Italy, improved clinical outcomes were predicted in the younger age group with NLR < 3. Severe clinical outcomes were observed in older patients with NLR > 4 [46]. Metacentric studies with large sample sizes should be conducted to eliminate potential biases.

Neutrophilia and lymphocytopenia are hallmarks of acute infection. Fox et al. noted neutrophil infiltration in the alveolar space, pulmonary capillaries, inflammation of mucosa with abundant neutrophils, and fibrin deposition in capillaries [47]. Barnes et al. proposed NETs as a potential mechanism for neutrophil-mediated damage [48]. Also, mechanical ventilation is required in severe COVID-19 with potential complications, as mechanical ventilation can itself cause alveolar damage [49]. Yildiz et al. noted NETs in mice models requiring mechanical ventilation [50].

Treatment strategies

Currently, the epidemic requires various combined drug therapeutics for severe COVID-19 cases and reduction of inflammation in every aspect of the inflammatory cycle and host response. Drugs targeting the neutrophilic egress and localization may reduce the clinical complications of alveolar damage and ARDS in COVID-19 cases. Earlier studies evaluated the role of Vitamin C infusion with limited benefits in the influx of granulocytes, activation, and NET formation. Clinical trials observed in vitro suppression of NET release by small peptides such as P140 [51]. Regulation of chaperone-mediated autophagy and macroautophagy by P140 has decreased inflammation in autoimmune disorders [52].

Important chemokine signaling of neutrophils’ migration and the release of NET in the CXCL8/CXCR2 axis are under exploration [53]. Therefore, the CXCL8/CXCR2 axis antagonists have been evaluated in clinical trials of various respiratory distress conditions in influenza, eczema, and chronic obstructive pulmonary disease (COPD). Preclinical and clinical studies have tested the safety of AZD 5069 (a selective CXCR2 antagonist) [54] and SCH527123 [55] in COPD and asthma. The drug effectively blocked neutrophil egress and subsequent inflammatory activities in COPD patients. Other similar studies noted reduced neutrophilia with the use of danirixin (a CXCR2 inhibitor) [56,57] and navarixin (MK-7123/SCH 527123) [58] in phase 2 clinical trials in influenza and COPD cases. MS-986253 (a CXCL8 blocking antibody) is under investigation in COVID-19 patients [59].

Clinical trials have evaluated the potential role of sivelestat sodium (neutrophil elastase inhibitors) in patients with COPD and acute lung injury [60]. Sivelestat has been used with oseltamivir effectively in swine flu patients [61]. Elastase, a protease, degrades the proteins present in the alveolar basement membrane (e.g., elastin, collagen, and fibronectin) and aggravates alveolitis [62]. Additional lung tissue damage occurs by proteolytic priming of the viral glycoproteins, enabling membrane fusion in the host. Apart from the proteolytic effect, elastase has an important prothrombotic and proinflammatory role in the pulmonary vasculature [63].

Animal models of myeloproliferative tumors have evaluated the efficacy of peptidyl arginine deiminase IV (PAD4) inhibitors in reducing NET pathologies [64]. Some PAD4 inhibitors are Cl-amidine, YW-56, and GSK484. PAD4 converts arginine to citrulline in histones and promotes chromatin unwinding and NET release [65].

Recombinant DNase 1 can dissolve the NETs and provide some therapeutic benefits in combined therapeutic regimes [66]. The degradation products thus generated may also have proinflammatory activity [67], possibly mitigating inflammation spread and more significant tissue damage. Thus, further studies are required to evaluate the potential role of DNAse in severe SARS-CoV-2 infections.

Metoprolol is a β1 blocker shown to suppress NET-associated damage in the gall bladder. It also inhibits neutrophilic migration, reduces platelet aggregation and inflammation, and reduces infarct size in myocardial infarction cases [68]. Blocking the IL-1b/IL-1R interaction using anakinra (an IL-1R inhibitor) has shown promising results in initial clinical trials [69], reducing COVID-19 severity.

The C5a-C5aR1 interaction is another potential target [70]. The C5a molecule binds to the C5aR1 receptor and regulates neutrophilic activation and recruitment. The serum levels of these complement receptors are directly proportional to COVID-19 severity [71]. Vlaar et al. noted that the administration of IFX-1 (vilobelimab), a monoclonal antibody against C5a, and Solris (eculizumab), a C5 blocking antibody, shows adequate efficacy in the management of moderate and severe COVID-19 cases [72].

Corticosteroids have an anti-inflammatory effect and can improve lung tissue injury and ARDS. Systemic steroids administered to manage SARS-CoV-2 infections reduced neutrophil burst and recruitment at inflamed sites [73]. The immune regulation by corticosteroids is advisable but controversial since corticosteroid treatment could delay the clearance of SARS-CoV-2 in respiratory secretion or serum due to its immunosuppressive action [74]. Glucocorticoids have been effectively used in the treatment of SARS. In patients with COVID-19 and ARDS, methylprednisolone minimizes the risk of mortality. Li and Ma investigated the role of methylprednisolone in ARDS cases and found an increased risk of death [75].

## Conclusions

NLR is an inflammatory marker and a proven diagnostic predictor of outcome and case severity in non-mild SARS-CoV-2 infections. With its wide availability, NLR assays can quickly assess a patient's condition and help the clinician alter treatments accordingly. Early stratification helps screen critically ill patients, as the higher the NLR value, the greater the need for support and care. Future studies should be conducted in different geographical settings to correlate outcomes with NLR and age, gender, hemodynamic variable, and comorbid conditions.
